# Efficient malachite green biodegradation by *Pseudomonas plecoglossicide* MG2: process optimization, application in bioreactors, and degradation pathway

**DOI:** 10.1186/s12934-023-02194-z

**Published:** 2023-09-21

**Authors:** Magda A. El-Bendary, Mariam E. Fawzy, Mohamed Abdelraof, Mervat El-Sedik, Mousa A. Allam

**Affiliations:** 1 Microbial Chemistry Department, Biotechnology Research Institute, National Research Centre, 33 Bohouth St., Dokki, Giza, Egypt; 2 Water Pollution Research Department, Environmental Research and Climate Change Institute, National Research Centre, 33 Bohouth st., Dokki, Giza, Egypt; 3 Dyeing, Printing and Textile Auxiliaries Department, Textile Research and Technology Institute, National Research Centre, 33 Bohouth st., Dokki, Giza, Egypt; 4 Spectroscopy Department, Physics Research Institute, National Research Centre, 33 Bohouth st., Dokki, Giza, Egypt

**Keywords:** Malachite green, Degradation, *Pseudomonas*, Optimization, Bioreactor, Cytotoxicity, Spectroscopic characterization, Degradation pathway

## Abstract

Microbial degradation of synthetic dyes is considered a promising green dye detoxification, cost-effective and eco-friendly approach. A detailed study on the decolorization and degradation of malachite green dye (MG) using a newly isolated *Pseudomonas plecoglossicide* MG2 was carried out. Optimization of MG biodegradation by the tested organism was investigated by using a UV–Vis spectrophotometer and the resultant degraded products were analyzed by liquid chromatography–mass spectrometry and FTIR. Also, the cytotoxicity of MG degraded products was studied on a human normal retina cell line. The optimum conditions for the significant maximum decolorization of MG dye (90–93%) by the tested organism were pH 6–7, inoculum size 4–6%, and incubation temperature 30–35 °C, under static and aerobic conditions. The performance of *Pseudomonas plecoglossicide* MG2 grown culture in the bioreactors using simulated wastewater was assessed. MG degradation (99% at 100 and 150 mg MG/l at an optimal pH) and COD removal (95.95%) by using *Pseudomonas plecoglossicide* MG2 culture were the best in the tested culture bioreactor in comparison with that in activated sludge or tested culture-activated sludge bioreactors.The FTIR spectrum of the biodegraded MG displayed significant spectral changes, especially in the fingerprint region 1500–500 as well as disappearance of some peaks and appearance of new peaks. Twelve degradation intermediates were identified by LC–MS. They were desmalachite green, didesmalachite green, tetradesmalachite green, 4-(diphenylmethyl)aniline, malachite green carbinol, bis[4-(dimethylamino)phenyl]methanone, [4-(dimethylamino)phenyl][4-(methyl-amino)phenyl]methanone, bis[4-(methylamino)phenyl]methanone, (4-amino- phenyl)[4-(methylamino)phenyl]methanone, bis(4-amino phenyl)methanone, (4-amino phenyl)methanone, and 4-(dimathylamino)benzaldehyde. According to LC–MS and FTIR data, two pathways for MG degradation by using *Pseudomonas plecoglossicide* MG2 were proposed. MG showed cytotoxicity to human normal retina cell line with LC_50_ of 28.9 µg/ml and LC_90_ at 79.7 µg/ml. On the other hand, MG bio-degraded products showed no toxicity to the tested cell line. Finally, this study proved that *Pseudomonas plecoglossicide* MG2 could be used as an efficient, renewable, eco-friendly, sustainable and cost-effective biotechnology tool for the treatment of dye wastewater effluent.

## Introduction

Extensive utilization of synthetic dyes is detrimental to the environment and human health. Discharging the dyes wastewater from different industries into natural water streams increases toxicity, the chemical oxygen demand of the effluent, and reduces light penetration, which affects the photosynthesis process. Dyes have a stable and difficult biodegradable structure and they are toxic, mutagenic and carcinogenic [40, 48].

MG dye is a dark green, crystalline solid, water soluble cationic dye (basic dye), named as N-methylated diaminotriphenylmethane [49]. The data about the chemical structure, molecular formula, molecular weight, λ_max_, etc. for MG are summarized in Table 1. MG is one of the most used dyes in several industries such as dyeing, papermaking, pharmaceuticals, cosmetics, etc. [36]. There are many reports about the cytotoxicity effect of MG against cells from different organism including humans. In addition, carcinogenesis, teratogenesis, and mutagenesis potential of MG were reported in the human cells [46]. Despite its high toxicity, genotoxicity and carcinogenicity, MG is currently used extensively worldwide for dyeing due to its relatively low cost [35].

**Table 1:** MG dye used and its synonymies, λmax, structure, molecular weight and formula

C.I. name, common name and Abbr	Wavelength λmax (nm)	Chemical structure, molecular formula and molecular weight
C.i. Basic green, Malachite green, Aniline green, Diamond green B, Victoria green B	620	
		MF: C_23_H_25_ClN_2_; MW: 364.911

Different physico-chemical and biological approaches have been used for the decolorization/degradation of dye wastewater. Physico-chemical methods such as coagulation, flocculation, adsorption, ion exchange, precipitation and photo degradation were successfully used [4, 14]. However, these methods have some drawbacks such as being economically unfeasible and producing sludge, which results in a secondary pollution. Nowadays, biological methods using microorganisms are an interesting, eco-friendly, cost-effective technology for the biotreatment of wastewater [2]. Different bacterial cultures showed high efficiency in MG decolorization/degradation such as *Kocuriarosea* MTCC 1532; *Sphingomonas paucinabilis*; *Brevibacillus laterosporus*; *Pseudomonas* sp. DY1; *Klebsiella terrigenaptcc*; *Ochrobactrum* sp JN214485; *Pseudomonas* sp. YB2; *Bacillus vietnamensis* sp. MSB17; *Pseudomonas veronii* JW3-6 and *Stenotrophomonas maltophilia* [5, 13, 20, 37, 38, 42, 43, 45].

In a previous study, *Pseudomonas plecoglossicide* MG2 (accession no. MN933934.1) was isolated and showed 79% MG decolorization after 48 h. It showed oxireductive enzyme activities, and the solution after degradation was non phytotoxic. The present study aims to optimize the MG degradation by *Pseudomonas plecoglossicide* MG2. Also, bioremediation of MG in simulated wastewater using three different bioreactors was investigated. In addition, the degradation pathway of the tested organism was studied. Finally, the cytotoxicity of the degraded products was assessed.

## Materials and methods

### Chemicals

MG (dye content 90%, MW 929, Abs (616–620) was purchased from Oxford. HPLC ethyl acetate and HPLC methanol were purchased from Sigma-Aldrich. All reagents and other chemicals were of high-purity analytical grade and purchased from Merk and Sigma-Aldrich.

### Microorganisms and growth conditions

**Fig. 1:**
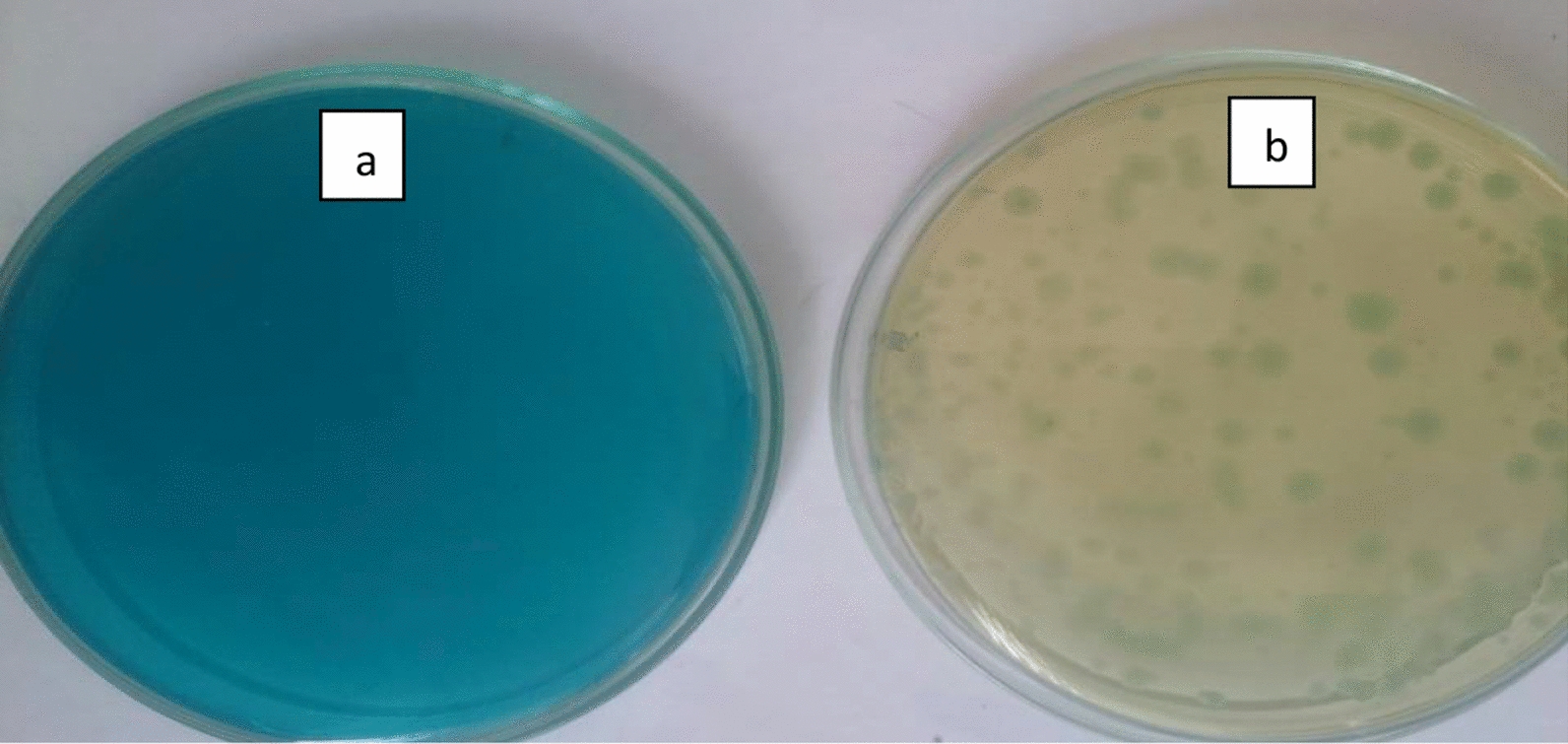
MG degradation by *Pseudomonas plecoglossicide* MG2. **a** MG control plate with 50 mg/l MG. **b** MG plate  after growth of *Pseudomonas plecoglossicide* MG2 for 72 h

*Pseudomonas plecoglossicide* MG2 was isolated from the sludge of dye industry effluent and deposited in GenBank with accession number of MN933934.1. It decolorized MG (Fig. 1) and showed oxireductive enzyme activities including laccase, lignin peroxidase, manganese peroxidase, triphenylmethane reductase, anthraquinone reductase and azoreductase. It was maintained on nutrient agar (NA) slants and stored at 4 °C.

### Malachite green dye decolorization assay

The isolated bacterium was grown in 50 ml nutrient broth medium (NB) amended with 50 mg/l of tested dye and incubated under shaking conditions (150 rpm) at 30 °C. Samples from each flask were withdrawn at different intervals (2–7 days), centrifuged at 8000 rpm for 10 min. A biotic dye control (without microorganism) was operated under the same conditions to be used as a blank. The decolorization of MG in the supernatant was evaluated using a UV–Vis spectrophotometer (Carry 100 Ultraviolet–visible spectrophotometer, Agilent, USA) at 620 nm. The decolorization percentage was calculated using the following equation according to Roy et al. [39]:

$${\text{Decolorization }}\left( \% \right) \, = \, \left( {{\text{A }}{-}{\text{ B}}} \right) \, /{\text{ A }} \times { 1}00$$

where, A and B are the absorbances of blank and the solution after bacterial dye decolorization, respectively.

### Effect of different growth media on decolorization efficiency

The quantitative determination of MG decolorization activity by *Pseudomonas plecoglossicide* MG2 was investigated using MS medium [45], MSB medium [5] and nutrient broth medium (NB). These media were supplemented with 25 mg/l of MG.

### Optimization of MG degradation by *Pseudomonas plecoglossicide* MG2

In order to determine the optimized cultural conditions that enhance the MG degradation by *Pseudomonas plecoglossicide* MG2, different physical parameters including, pH (4–9), temperature (25–45 °C), inoculum size (2–10%) and medium volume (20–80 ml/250 ml Erlenmeyer flask) were studied. The effect of initial dye concentration in the medium (25–400 mg/l) on the MG degradation was also evaluated. Effect of shaking and static conditions were studied. MG degradation activity in each experiment was evaluated by withdrawing samples at different intervals from 24 to 144 h. In each experiment, the medium without the tested organism was used as a control.

### Bioreactors performance

#### *Simulated wastewater composition*

Simulated stock wastewater was prepared according to Hait and Mazumder [17] and it composed of /l: dextrose, 10 g; NH_4_NO_3_, 2.857 g; KH_2_PO_4_, 0.894 g; K_2_HPO_4_, 43.5 mg; MgSO_4_7H_2_O, 45 mg; FeCl_3_6H_2_O, 0.5 mg; Na_2_HPO_4_7H_2_O, 66.8 mg; NH_4_Cl, 3.4 mg and CaCl_2_, 55 mg. The chemicals contain carbon source with micro and macro nutrients dissolved in tap water. The prepared stock solution was diluted with tap water to get varying COD concentration.

Decolorization of different MG concentrations (50, 100 and 150 mg/l) were tested in simulated wastewater bioreactors.

#### *Reactor set-up*

Three laboratory Plexiglas’s columns operated in batch mode were applied for MG- simulated wastewater. Each column is cylindrical in shape with a diameter of 7 cm and a length of 90 cm and a total volume of 2 L. Air is introduced into laboratory columns using air pumps with fixed flow rates to keep the biomass in a complete mixing condition. Three experimental runs were carried out at room temperature (25 ± 2 °C) simultaneously. The columns were inoculated with i- Activated sludge (AS) with 3–4 g/l, ii- Mixture of tested culture and activated sludge (TC-AS) (1:1), iii- Tested culture (TC) with 3–4 g/L. The air is turned twice a day and settling is allowed for 1 h, then the supernatant is drained for analysis and refilled with a fresh MG-simulated wastewater sample.

#### *Acclimatization and start-up*

The column operated by AS was seeded with mixed liquor suspended solids (MLSS) delivered from Zenin Wastewater Treatment Plant, Giza, Egypt. At the same time, the second column was inoculated with a mixture of *Pseudomonas plecoglossicide* MG2 and activated sludge with a ratio 1:1 (TC-AS). The third column was inoculated with *Pseudomonas plecoglossicide* MG2 (TC). Three experimental runs were operated using known concentrations of MG namely 50, 100 and 150 mg/l. The columns were fed with simulated wastewater and gradually different concentrations of MG were added to prevent the toxic effect of dye on the biomass and maintain the growth of cultures. Then the columns were operated continuously for 24 h until constant removal rates of COD were achieved. Finally, after reaching the steady state conditions, growth rate experiment was carried out to evaluate the optimum time needed for the biodegradation of MG.

#### *Analysis*

After reaching the steady state conditions, simulated wastewater and treated effluent from each experimental run at known dye concentration were characterized according to the Standard Methods for the Examination of Water and Wastewater [7]. The analysis includes pH, color, turbidity, chemical oxygen demand (COD), oxidation reduction potential (ORP) and dye concentration. Sludge samples were analyzed for total suspended solids (TSS) and volatile suspended solids (VSS) and undergo microscopic examination. COD was measured according to dichromate method using digestion for two hours at 150 °C on a Lovibond digestor then cooled followed by colorimetric measurement on a spectrophotometer.

The absorbance of malachite green was measured using a UV/Vis spectrophotometer at 620 nm. Oxidation reduction potential was measured by a waterproof multiparameter meter Model Hanna HI 98195. Turbidity was measured by turbidimeter Model palintest micro 950, pH was measured using a benchtop pH meter Jenway 3510 and color was measured by Lovibond spectrodirect.

#### MG degradation pathway by *Pseudomonas plecoglossicide* MG2

##### *Extraction of MG degradation products*

After complete MG degradation (100 mg/l) in the tested culture bioreactor, the solution was centrifuged at 8000 rpm for 10 min and the supernatant was extracted thrice with HPLC analytical grade ethyl acetate. The obtained extract was dryed in a rotary evaporator then dissolved in HPLC grade methanol for LC–ESI–MS, FTIR, and cytotoxicity studies.

##### *LC–ESI–MS*

LC–ESI–MS positive and negative ion acquisition mode were carried out on a XEVO TQD triple quadruple instrument. Waters Corporation, Milford, MA01757 U.S.A, Mass Spectrometer. All solvents and chemicals used are of HPLC analytical grade. Column: AC QUITY UPLC—BEH C18 1.7 µm-2.1 × 50 mm.

Column flow rate: 0.2 ml/min, solvent system: consisted of (A) water containing 0.1% formic acid (B) methanol containing 0.1% formic acid. The sample (100 μg/ml) solution was prepared using methanol, filtered using Polytetrafluorethylene (PTFE) membrane filter, 0.2 μm (then subjected to LC–ESI–MS analysis. Sample injection volumes (10 μl) were injected into the UPLC instrument equipped with a reverse phase C-18 column (AC QUITY UPLC—BEH C18 1.7 µm particle size—2.1 × 50 mm column). A sample mobile phase was prepared by filtering using 0.2 μm PTFE membrane filter and degassed by sonication before injection. Mobile phase elution was made with a flow rate of 0.2 ml/min using gradient mobile phase comprising two eluents: eluent A is H_2_O acidified with 0.1% formic acid and eluent B is methanol acidified with 0.1% formic acid. Elution was performed using the above gradient. The parameters for analysis were carried out using negative ion mode as follows: source temperature 150 °C, cone voltage 30 eV, capillary voltage 3 kV, desolvation temperature 440 °C, cone gas flow 50 l/h, and desolvation gas flow 900 l/h. Mass spectra were detected in the ESI negative ion mode between *m/z* 100–1000. The peaks and spectra were processed using the Mas lynx 4.1 software and tentatively identified by comparing their retention time (Rt) and mass spectrum with reported data.

##### FTIR

The FTIR spectra of MG samples before and after biodegradation were recorded by a JASCOW FT/IR-4700 spectrometer, Japan. The spectra were collected at a resolution of 4 cm^−1^ and a number of scans of 16 in the mid-infrared region of 4000–400 cm^−1^ using the ART technique. Samples of MG and its biodegraded products were directly applied on the diamond crystal of the ATR unit and then analyzed.

##### Cytotoxicity

The cytotoxicity of MG solution before and after bacterial degradation was tested in the Bioassay-Cell Culture Laboratory at National Research Centre on human normal retina cell line (RPE1). Cultivation of cells was carried out in cell culture flasks (75 cm^2^), using minimal essential medium containing Eagle’s salts (MEM) supplemented with 5% fetal bovine serum [(FBS), Gibco], 1% antibacterial and antifungal mixture (penicillin G, 100 U/ml; streptomycin sulfate, 100 µg/ml; amphotericin B, 25 µg/ml) and incubated at 37 °C under 5% CO_2_ humidified atmosphere using a carbon dioxide incubator (Sheldon, TC2323, Cornelius, OR, USA). Cells were batch cultured for 10 days. The cells were seeded in 96-well microtiter plates at concentration of 10 × 10^3^ cells/well. These plates were incubated at 37 °C for 24 h in CO_2_ incubator. After 48 h incubation, the medium was aspirated, fresh medium was added and cells were incubated without MG (negative control) or with MG solutions before and after bacterial degradation and incubated for an additional 48 h at 37 °C. Cell viability was assessed by colorimetric method, depending on the mitochondrial dependent reduction of yellow MTT (3-(4, 5-dimethylthiazol-2-yl)-2, 5- diphenyl tetrazolium bromide) to purple formazan [33]. So, the culture medium was aspirated and 100 µl of MTT solution (5 mg/ml) was added to each well and was further incubated for 4 h at 37 °C under 5% CO_2_ to allow formazan formation. After removal of MTT, 50 µl dimethyl sulfoxide (DMSO) was added to each well, and then the plates were further incubated for 30 min at 37 °C for solubilization of formazan crystals. The absorbance was read at 595 nm.

Percentages of cell viability were calculated as the following:

Cell viability % = (absorbance of treated cells/absorbance of control cells) × 100.

## Results and discussion

Due to the high efficiency and low price of MG, it is utilized in different industrial sectors. However, excessive accumulation of MG causes severe toxicity to environment and human health. MG degradation using different chemical and physical treatments have several limitations involving the resultant toxic products, slow, and high cost [20]. Therefore, an alternative treatment by using microbial degradation has currently attracted attention due to its low cost, green, and fast approach [5].

### Effect of growth media on the MG degradation

**Fig. 2:**
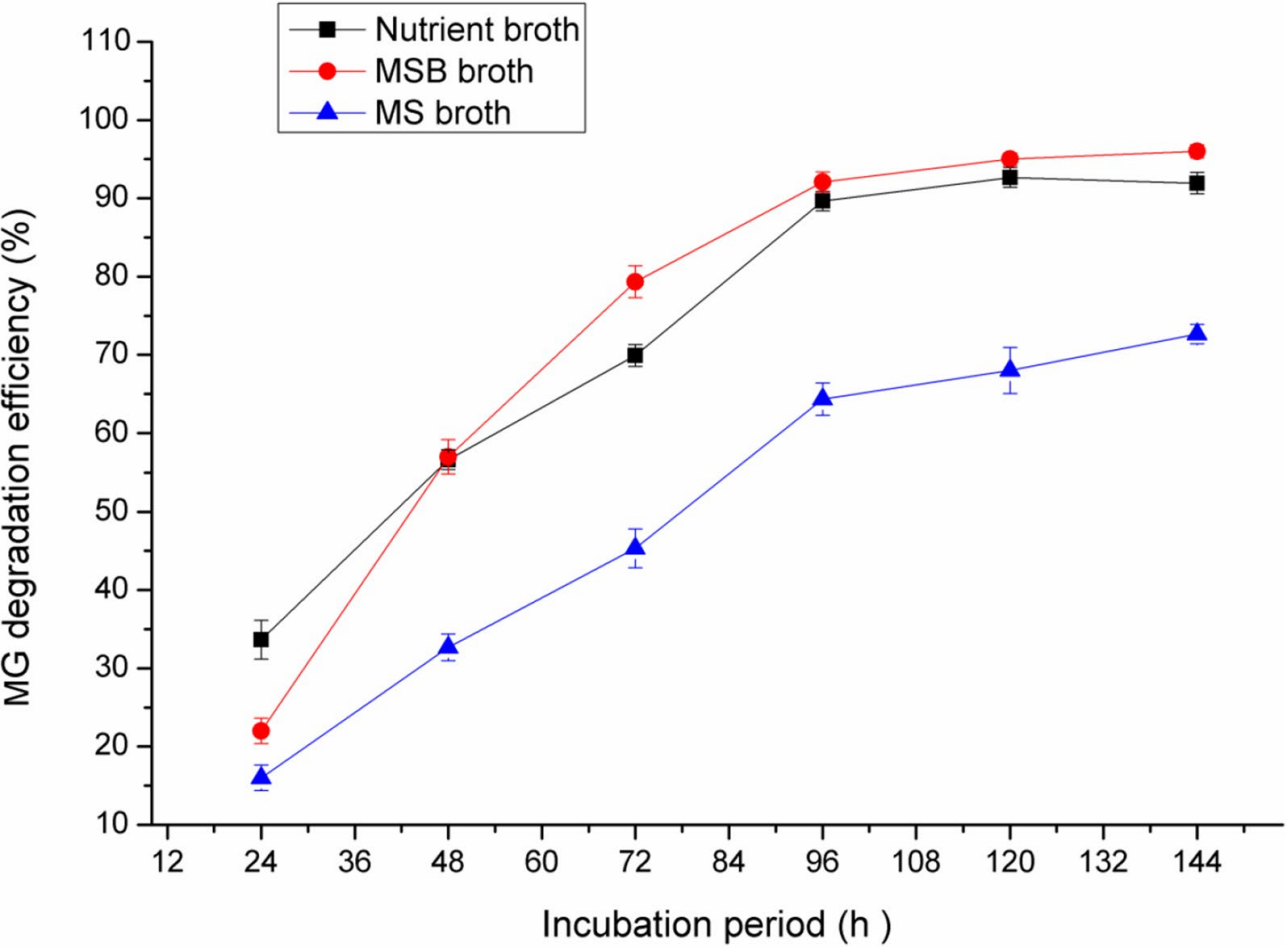
Effect of different culture media on MG degradation efficiency by *Pseudomonas plecoglossicide* MG2

As shown in Fig. 2, the maximum MG decolorization by *Pseudomonas plecoglossicide* MG2 was achieved with MSB and this result is nearly close to that obtained with NB (near to 90% decolorization after 96 h incubation), while MS medium showed about 59% decolorization after the same incubation period. Therefore, MSB was selected for further optimization of decolorization activity by *Pseudomonas plecoglossicide* MG2. MSB and NB media contain peptone in their contents. Peptone is a mixture of water soluble compounds resulting from the enzymatic or acid hydrolysis of proteinaceous substances. It provides the culture medium with amino acids, peptides and carbohydrates that are necessary for microbial growth and enzyme production [27].

There are many reports about MG decoloration and degradation by bacteria. Abu-Hussien et al. [3] reported that *Streptomyces exfoliates*, *Tenacibaculum* spp. HMG1, *Pseudomonas* spp. YB2, strain S20 and *Enterococcus* sp. could efficiently decolorize 96% of 100 mg/l MG after 120 h, 98.8% of 20 mg/l MG in 12 h, 100% of 1000 mg/l MG in 12 h, 98% of 300 mg/l MG after 96 h, and 94% of 20 mg/l MG, respectively. While *Bacillus cereus* and *Pseudomonas aeruginosa* showed 16% and 19% MG decolorization, respectively [3].

Song et al. [42] isolated *Pseudomonas veronii* and it exhibited 93.5% degradatoion of 50 mg/l MG*.* Also, He et al. [18] isolated a *Pseudomonas* sp. strain and it showed about 80% MG degradation (50 mg/l) in 14 h. All these experiments were performed in microbial growth medium, however, the same degradation potentials of tested microorganisms could not be achieved in the natural or simulated environment.

In the present study, the MG in simulated wastewater bioreactors was efficiently degraded by *Pseudomonas plecoglossicide* MG2 and the degradation products showed no cytotoxicity as will be mentioned later.

### Optimization of MG degradation by *Pseudomonas plecoglossicide* MG2

Effect of several parameters that can contribute to MG decolorization efficiency by *Pseudomonas plecoglossicide* MG2 such as pH, temperature, inoculum size, aeration, medium volume and different MG concentrations were evaluated during the incubation period of 24–144 h.

### *Effect of pH*

**Fig. 3:**
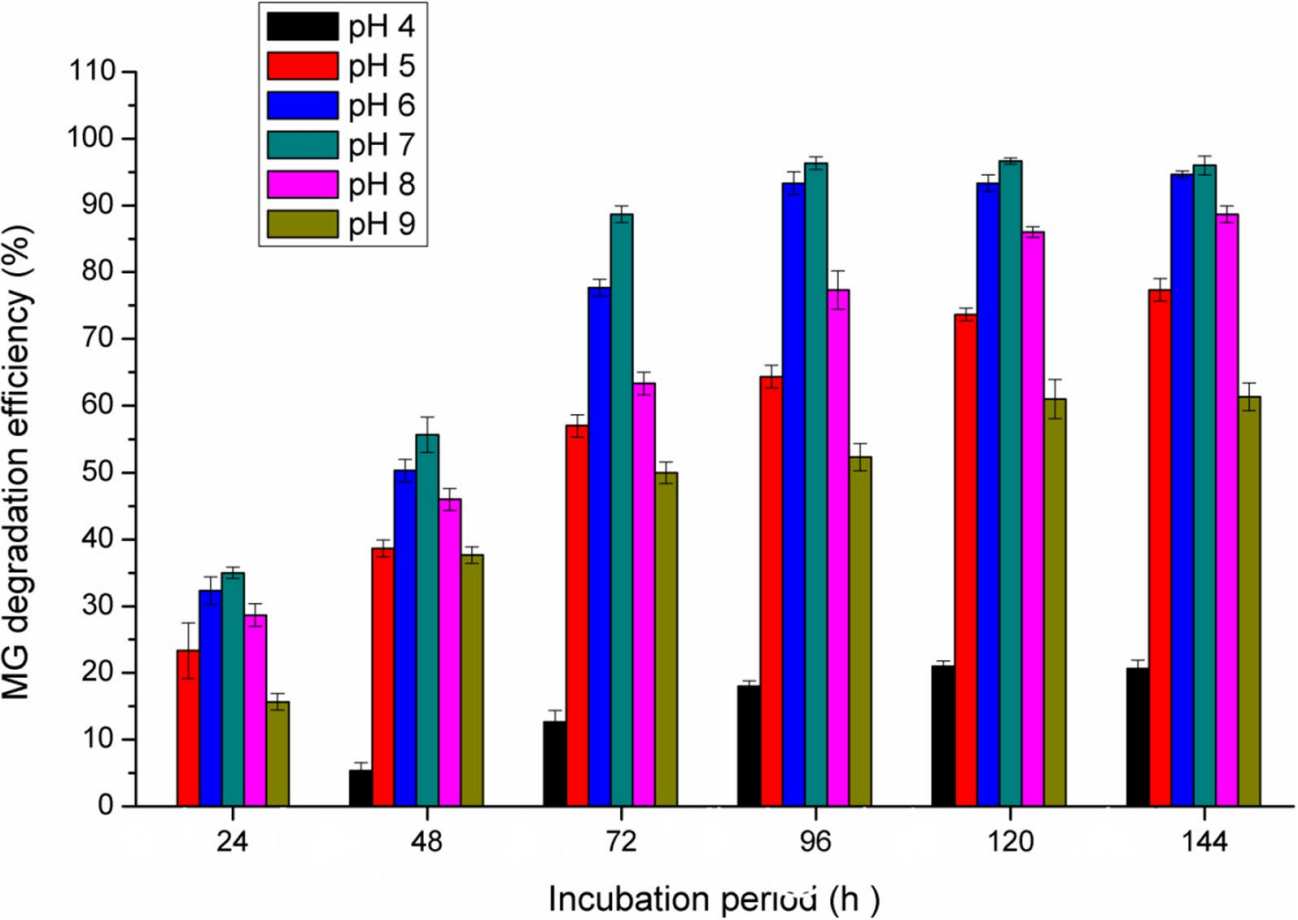
Effect of pH on the MG degradation efficiency by *Pseudomonas plecoglossicide* MG2

The dye degradation at higher pH values is important especially for industrial effluent bioremediation, which is carried out under basic conditions. Usually, the pH level had a significant effect on the dye degradation, since the optimum pH for the maximum decolorization of dyes is between 6 and 10 [21]. In the present study, Fig. 3 shows the maximum decolorization activity was noted at pH 6–7 with the maximum decolorization at pH 7 (92.5% degradation after 96 h). In contrast, at pH 4 the decolorization efficiency was sharply decreased. However, at pH 5 and pH 8 a valuable MG degradation was obtained (61%, and 74.5%, respectively after 96 h).

Similarly, Vijayalakshmidevi and Muthukumar [45] reported that the maximum MG decolorization using *Ochrobactrum* sp JN214485 was at pH 6. Also, Du et al. [13] found that *Pseudomonas sp. strain DY1* efficiently decolorized MG at pH 6.6. In the study of Song et al. [42], they found that the highest MG decolorization by *Pseudomonas veronii* JW3-6 was at pH 5–7 with the maximum activity at pH 7. In accordance with these results, the highest MG decolorization by *Stenotrophomonas maltophilia* was at pH 6–7 [5]. Tao et al. [43] reported that the MG decolorization was not pH dependent and complete decolorization was obtained across a wide pH range from 5 to 9 by *Pseudomonas* sp. YB2.

The degradation efficiency at pH 4 and 9 was proved to be not significant, which reflects the unfavorable highly acidic and alkaline conditions for the microbial growth. Also, it is known that the pH value of the medium dominates the transport of dye molecules across the cell membrane, which is considered the rate limiting step in decolorization process [23].

### *Effect of temperature*

The incubation temperature affects both microbial growth and enzyme activities, and thus affects the rate of dye decolorization [23].

**Fig. 4:**
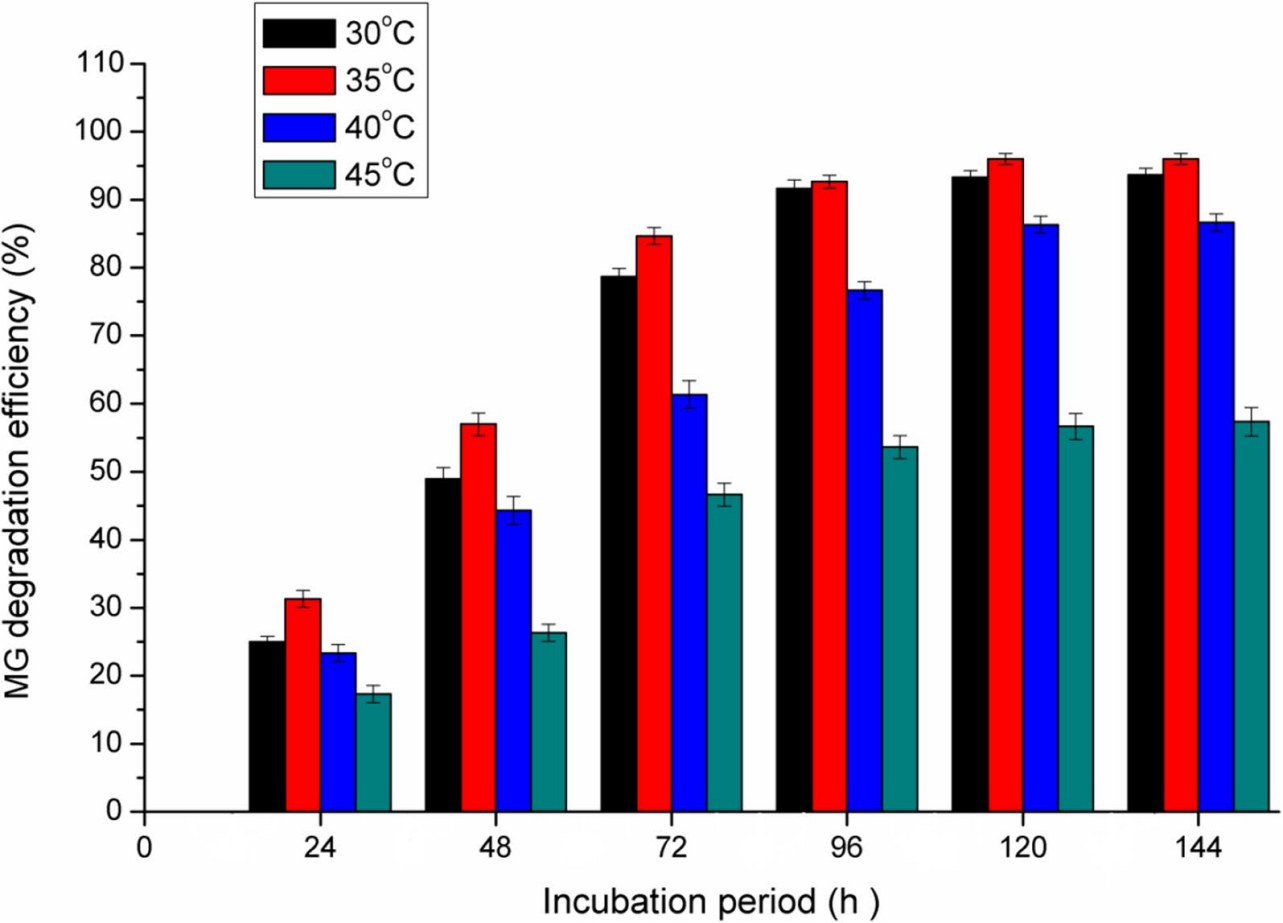
Effect of temperature on MG degradation by *Pseudomonas plecoglossicide* MG2

MG degradation efficiency by *Pseudomonas plecoglossicide* MG2 was investigated under different temperatures as shown in Fig. 4. The results demonstrated that the maximum decolorization activity was found between 30 and 35 °C with maximum decolorization at 35 °C (91.5% after 96 h). A valuable decolorization percent was obtained at 40 °C. Meanwhile, lower decolorization activity was observed at 45 °C (56%). The decline decolorization activity at higher temperatures can be attributed to the loss of cell viability, decreased rates of enzymes production or the denaturation of enzymes that are responsible for MG decolorization [21, 23]. MG was efficiently decolorized (90–97%) at 28–30 °C using *Pseudomonas sp. strain DY1* as reported by Du et al. [13]. It was reported that MG decolorization by *Pseudomonas veronii* JW3-6 was more active (92.9%) at 30 °C and subsequently reduced to 70.1% at 40 °C [42]. Alaya et al. [5] concluded that the temperature range 25–30 °C was suitable for MG decolorization by *Stenotrophomonas maltophilia* as compared to temperatures higher than 35 °C. In addition, the maximum rate of MG decolorization is generally observed related to the optimum cell growth of *Pseudomonas* sp. YB2 [43] and *Ochrobactrum* sp JN214485 [45] at 30 °C.

### *Effect of inoculum size*

**Fig. 5:**
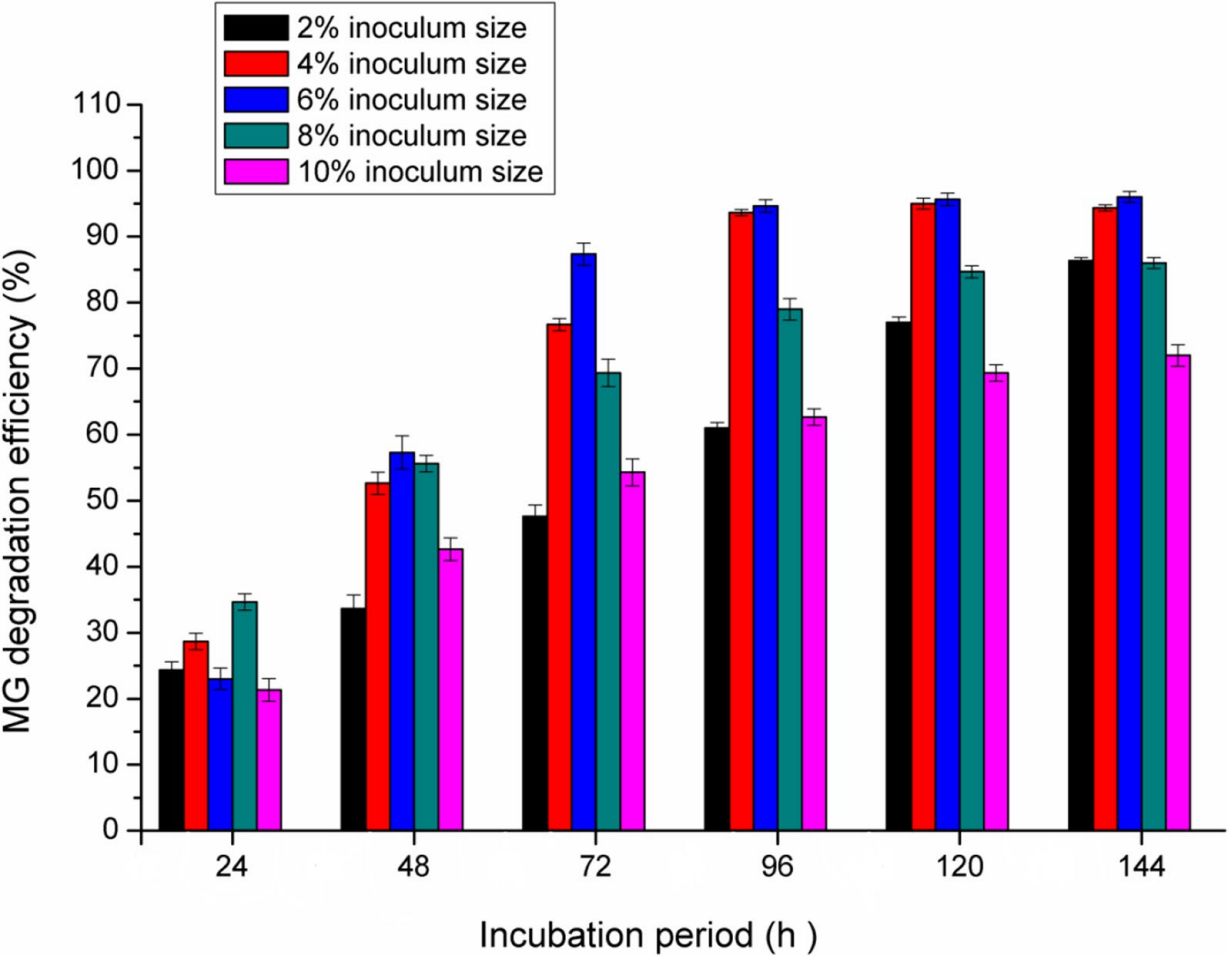
Effect of inoculum size on MG degradation efficiency by *Pseudomonas plecoglossicide* MG2

The inoculum concentration of *pseudomonas plecoglossicide* MG2 directly affects the MG decolorization. As shown in Fig. 5, at 24 h incubation, the best inoculum size is 8% (34% MG degradation). At 48–72 incubation, the increase in inoculum concentration from 2 to 6% lead to a gradual increase in MG decolorization with the maximum degradation at 6% inoculum size (84%). At 96–144 h incubation, the MG decolorization efficiency was the same using inoculum size 4 and 6% (about 90%). It was noted that higher inoculum size than 6% resulted in lower MG decolorization activity at 48–144 h incubation (degradation reduction about 41–80%). This could be related to the restraint of bacterial metabolic activities at a higher density of cells due to nutrient limitations and lower O_2_ transfer [1]. At lower inoculum size (2%), MG decolorization decreased and this may be related to longer time for cells to multiply and lowering the enzyme production. In this way, Kabeer et al. [20] reported that *Bacillus vietnamensis* sp. MSB17 could effectively decolorize MG using 4% inoculum size. More than this inoculum size lead to constant reduction of MG degradation.

### *Effect of aeration*

**Fig. 6:**
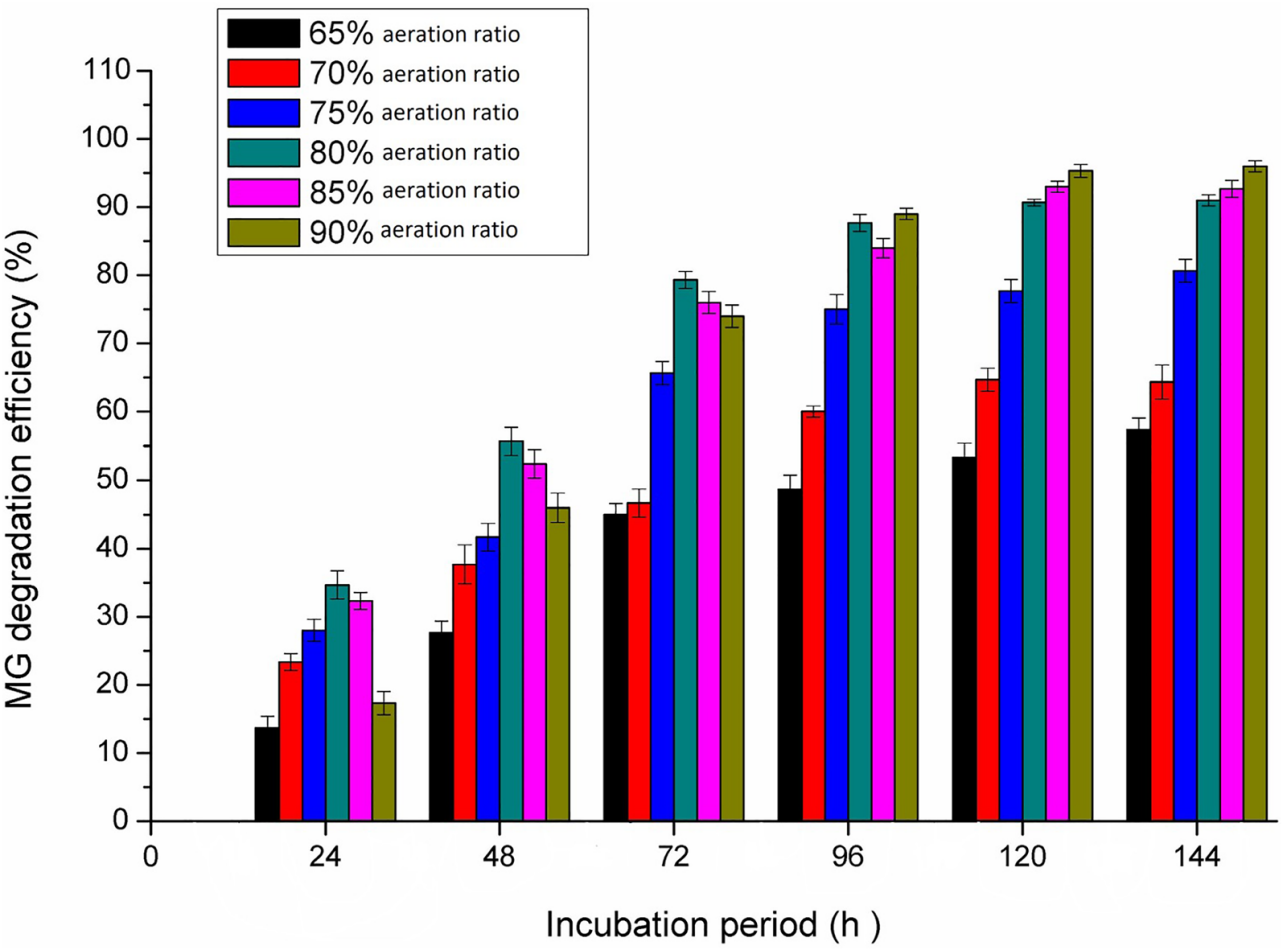
Effect of aeration on the degradation efficiency of *Pseudomonas plecoglossicide* MG2

Under aerobic conditions, oxygen transfer in the culture medium plays an important role in each of the cell growth and product formation. As illustrated in Fig. 6, the MG decolorization gradually increased with increasing the aeration levels from 65 to 80%. The maximum MG decolorization by *Pseudomonas plecoglossicide* MG2 was obtained at the highest aeration ratio (80–90%) at 96 h and a longer incubation period. The observed decrease of MG decolorization at lower aeration ratios is due to oxygen transfer limitations, leading to a decrease in the enzymatic production by *Pseudomonas plecoglossicide* MG2. Tao et al. [43] reported that *Pseudomonas* sp. YB2 efficiently decolorized MG at high aeration ratio in 250 ml Erlenmeyer flask; however the growth decreased when the solution volume increased in the flasks (low aeration ratio) and subsequently the MG decolorization activity decreased.

**Fig. 7:**
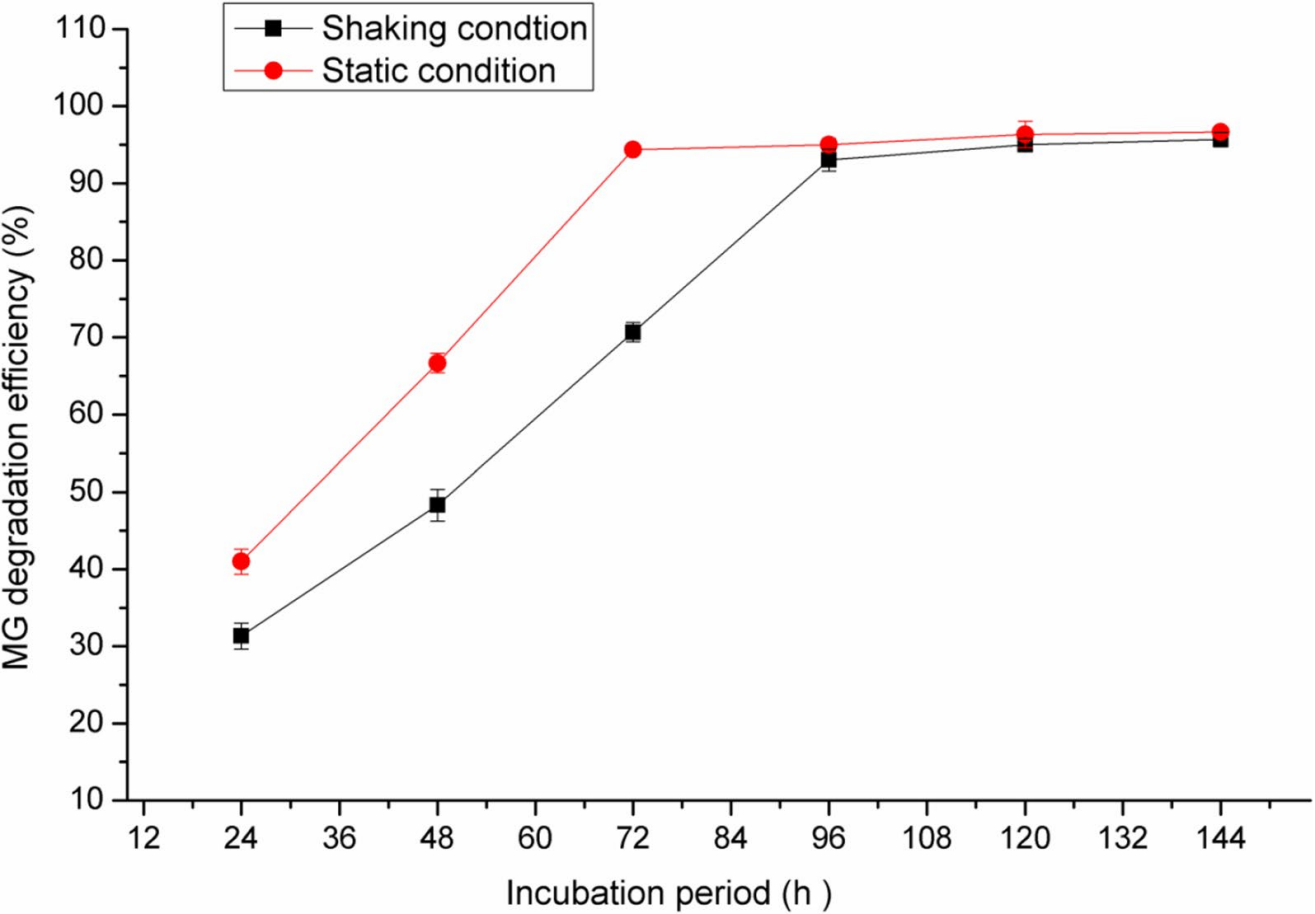
Effect of shaking and static conditions on MG degradation efficiency of *Pseudomonas plecoglossicide* MG2

In the present study, the effect of static and shaking conditions were also studied and the results are illustrated in Fig. 7. The decolorization of 50 mg/l of MG was obtained after 72 h with degradation percent of 94% under static condition whereas it was 65% after the same period under shaking condition. Increasing the incubation period to 96 h under shaking condition resulted in the maximum MG decolorization (92%).

In a previous study, *Pseudomonas plecoglossicide* MG2 proved the production of multi-oxidative-reductive enzymes such as laccase, lignin peroxidase, manganese peroxidase, tyrosinase, triphenylmethane reductase, anthraquinone reductase and azoreductase. Some of these enzymes switched on under aerobic conditions and others switched on under static conditions. Therefore, this organism can decolorize MG under shaking and static conditions.

Khan et al. [21] reported that the MG decolorization was observed significantly under static condition which could be attributed to the higher activity of reductive enzymes. Moreover, a sufficient amount of oxygen is also required for the activity of oxidative enzymes that are available under the static conditions. However, the slow decolorization reaction under shaking conditions was due to high oxygen amount that inhibited the reductive enzymes [11]. It was suggested that, MG decolorization under static conditions by microorganisms is related to the availability of reduced electron carriers such as NADH to reduce MG. Interestingly, most of MG decolorization strains demonstrated high efficiency under static or anaerobic condition as reported by Parshetti et al. [37] and Chaturvedi and Verma [9]. *Pseudomonas plecoglossicide* MG2 had also higher MG degradation efficiency under shaking conditions, which could support its practical application due to its ease of operation [43]. Alaya et al. [5] reported that the decolorization of MG was stimulated by incubation of *Stenotrophomonas maltophilia* under shaking conditions with increasing the oxygen levels in the liquid medium.

### *Effect of initial dye concentration*

**Fig. 8:**
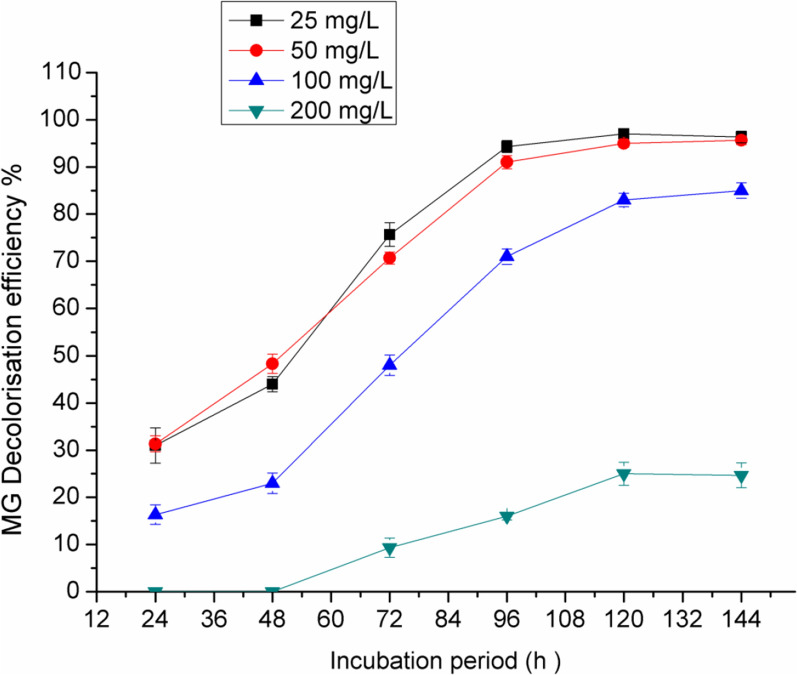
Effect of initial MG concentration on its degradation efficiency by *Pseudomonas plecoglossicide* MG2

Degradation of MG was performed with different initial concentrations (25–200 mg/l). As shown in Fig. 8, *Pseudomonas plecoglossicide* MG2 showed high efficiency in MG degradation at MG concentrations of 25–100 mg/l with nearly complete degradation after 96 h for 25–50 mg/l MG (94.7%) and 120 h for 100 mg/l MG (81.5%). Meanwhile, at 200 mg/l MG, the degradation percent decreased to about 18.9% even after 144 h incubation. Moreover, the bacterial density in the culture medium was remarkably decreased and the degradation efficiency was reduced to about 8% at 400 mg/l MG after incubation for 144 h (data not shown). With a further increase in MG concentration greater than 400 mg/l, the decolorization was negligible, indicating the inhibition effect of high MG concentration on the growth and activity of the tested organism.

Tony et al. [44] suggested that the decolorization rate decreased gradually as dye concentration increased. This reduction might be related to the toxic effect of dye on bacterial cells or the incorrect binding of the dye molecules with the enzyme active sites. In accordance with these results, MG at higher concentrations could inhibit the growth of *Kocuriarosea* MTCC 1532 [37], *Pseudomona veronii* JW3-6 [42] and *Bacillus vietnamensis* MSB17 [20].

### Bioreactor

**Fig. 9:**
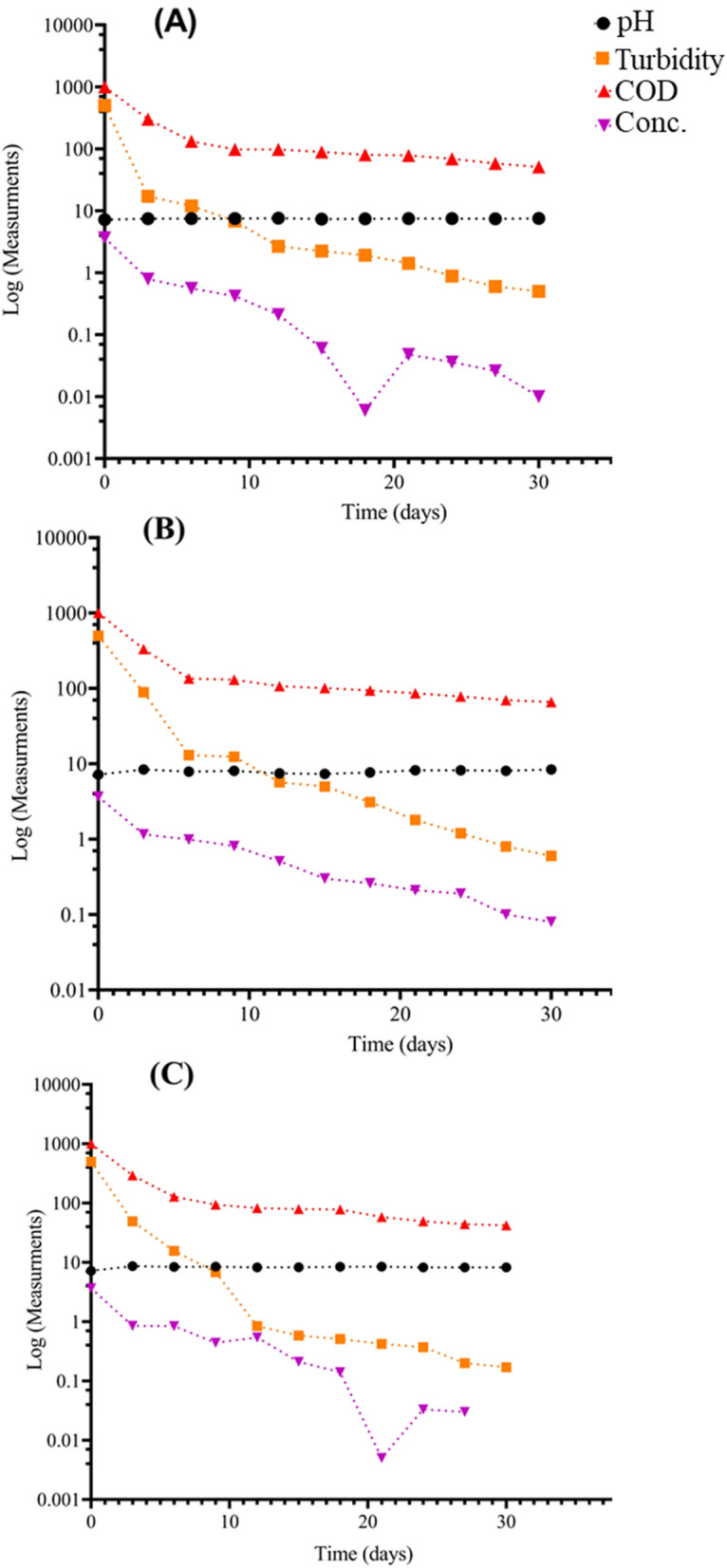
Variation of different pollution parameters at 50 mg/l MG for the different bioreactors. **A**, **B** and **C** are, activated sludge, tested culture-activated sluge, tested culture bioreactors, respectively

**Fig. 10:**
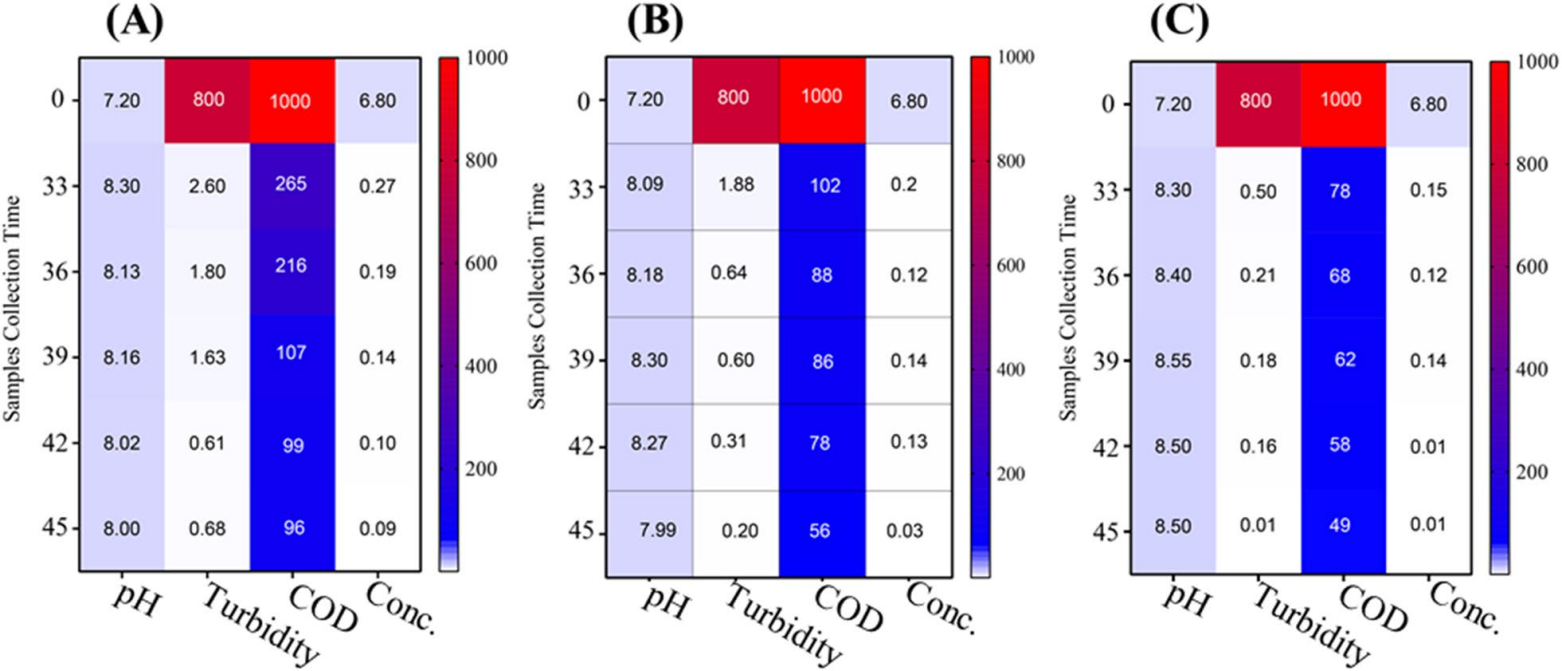
Heatmap showing the performance of different bioreactors at 100 mg/l MG. **A**, **B** and **C** are activated sludge, tested culture-activated sluge, tested culture bioreactors, respectively

**Fig. 11:**
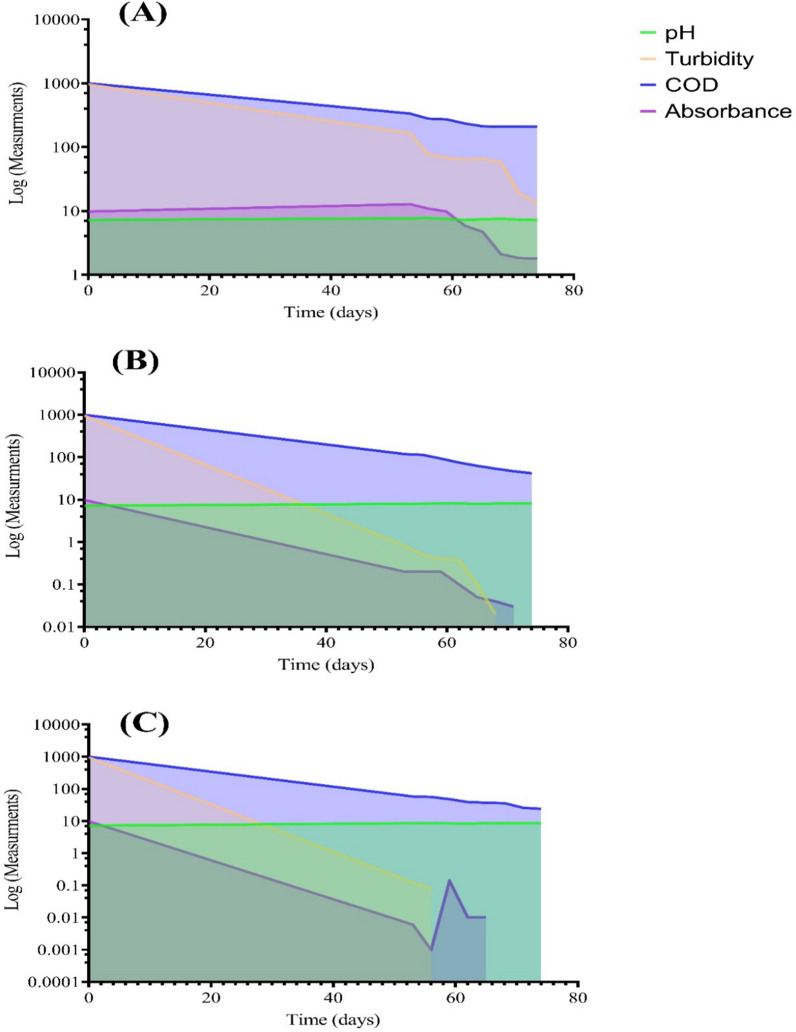
Area plot showing the performance of different bioreactors at 150 mg/l of MG. **A**, **B** and **C** are activated sludge, tested culture-activated sluge, tested culture bioreactors, respectively

The results depicted in Figs. 9, 10, 11 showed the performance of AS, TC-AS and TC bioreactors at different concentrations of malachite green (50, 100 and 150 mg/l).

### *Effect of simulated wastewater pH on MG decolorization*

The pH of wastewater plays an important role in dye decolorization. It was reported that bacterial strains have a favorable pH 7–8 for decolorization [34].

The effect of AS on MG decolorization at different dye concentrations was achieved at an optimal pH varied from 7.3 to 7.6 at 50 mg/l MG, 8–8.3 at 100 mg/l and 7.2 − 7.8 at 150 mg/l. The results showed that high decolorization for AS was achieved at 100 mg MG/l with an average percentage removal of 98.3% at a pH ranged from 8 to 8.3. Increasing the concentration of dye up to 150 mg/l leads to decrement of the decolorization percentage to 36.5%. Chen [10] reported that using conventional AS was not efficient for decolorization of dyes at high concentrations. On the other hand, it was noticed that the TC-AS decolrization efficiency was almost the same at 100 mg MG/l and 150 mg/l reached a value greater than 98% at a pH ranged from 7.9 to 8.3. However, TC showed the highest dye decolorization efficiency with an average value exceeding 99% at 100 and 150 mg MG/l at an optimal pH ranged from 8.3 to 8.6. These results are in agreement with the data reported by Mahmoud et al. [28]. Shah [41] found that using the isolated pure strain either alone or mixed with AS increase the decolorization of MG in the presence of carbon source. The cell surface was improved to be negatively charged and leads to more ionic attraction leading to a high binding affinity of MG molecules [34]. It was reported that at alkaline medium (pH > 7) the uptake of MG increased due to the change in polarity [31]. In the present work, the obtained results as illustrated in Figs. 9, 10, 11 showed clearly that MG removal increased as pH of the solution increased and the best decolorization occurred at alkaline medium (pH > 7.9).

### *Effect of dye concentration on organic matter removal*

Decolorization of MG is mainly affected by dye concentration [15]. Figures 9, 10, 11 show the variation of COD concentration with time (days) at different dye concentrations. Effect of different concentrations of MG were examined starting from 50 mg/l, then increased to 100 mg/l and finally 150 mg/l on the degradation rate of MG in simulated wastewater using different bioreactors. It was obvious that at the lowest MG concentration (50 mg/l), TC achieved the best removal efficiency for COD compared to AS and TC-AS in the order of TC > AS > TC-AS. While increasing the concentration up to 100 mg/l, the performance was in the order of TC > TC-AS > AS. Moreover, reaching to 150 mg/l, the removal efficiency for organic matter was in the order of TC > TC-AS > AS. It is worth to mention that AS was deteriorated and reached 75.43% removal efficiency.

At 50 mg/l of MG, the AS reached a removal rate of almost 89.31% with an average COD removal value of 105.3 mgO_2_/l. This can be attributed to the capability of AS under aerobic conditions to produce certain enzymes such as manganese peroxidase and laccase which degrade MG to CO_2_ and water resulted in an increase of MLSS [16]. In this study, it was observed that the removal rate of COD by AS dropped from 89.31 to 75% with increasing the concentration of MG from 50 to 150 mg/l. This may be due to high MG concentrations caused incorporable impact on MLSS resulting in a change of AS characteristics [30]. As well as, at high MG concentrations, the surface of AS (aerobic granules) became sutured with dye [24]. It also attributed to the adsorption of MG to the sludge granules and the oxygen uptake rate decrement of biosorption [29].

The results proved that incorporation of AS with the pure tested culture at a concentration of MG above 50 mg/l have a better removal rates for organic matter than the AS due to the synergetic interaction between different microorganisms [12, 22]. The average percentage removal for COD increased up to 92.95%. Furthermore, raising up the concentration to 150 mg/l did not significantly affect the performance of organic matter removal (92.41%). Song et al. [42] studied the use of *pseudomonas veroni* Jw-3–6 for the degradation of 50 mg/l of MG within a period of 6 days and reached 93.5%. The results of this study were much better than those obtained by Nath et al. [34] who obtained a removal value of 79% for COD and dye removal of 96% using AS at 50 mg/l at a hydraulic retention time of 72 h. The efficiency of TC at 50 mg/l was 90.53% and at 100 mg/l reached 94.47%. Finally the results of increasing the dye concentration to 150 mg/l indicated that the tested strain bioreactor was capable of biodegradation of MG efficiently. The residual COD reached 24 mgO_2_/l with an average COD removal rate of 95.95%. It was clear that the tested culture could survive at high dye concentrations and achieve the best removal efficiency for organic matter.

### *Optimum time for MG biodegradation at different dye concentrations*

The steady state conditions were reached for all the tested bioreactors after 26 days at 50 mg MG/l. Raising the concentration up to 100 mg/l the adaptation time decreased to 20 days and after more than 18 days it was completely steady for the degradation of 150 mg/l MG. After that the growth rate experiments revealed that the time needed for biodegradation of MG dye was different for each bioreactor at different dye concentration. For AS bioreactor, the time needed at 50 and 100 mg/l was almost 8 h while at 150 mg/l, it reached up to 12 h which is better than that achieved by Nath et al. [34]. The TC-AS bioreactor decreased the time needed for biodegradation in comparison with AS bioreactor at different concentrations of MG. Starting with 50 mg/l it takes 4 h and 8 h for both 100 and 150 mg/l, respectively. However, for the TC bioreactor it took only 4 h to reach complete degradation at all the examined dye concentrations. Table 2 shows the minimum and maximum COD removal values at different MG concentrations at the optimum time of biodegradation.

**Table 2:** Minimum, maximum and average COD removal rates at different dye concentrations

Bioreactor	50 mg/l			100 mg/l			150 mg/l		
	Min	Max	%R	Min	Max	%R	Min	Max	%R
AS	51	58	94.55	68	71	93.05	209	210	79.05
TC-AS	66	70	93.2	49	51	95.00	42	54	93.10
TC	42	44	95.7	38	45	95.85	24	36	97
Order of performance	TC > AS > TC-AS			TC > TC-AS > AS			TC > TC-AS > AS		

### *Biomass of different cultures*

The results obtained for the biodegradation of MG using AS revealed that the MLSS and MLVSS decreased with increasing the dye concentration. The MLSS was 3.9, 3.21 and 3.54 g/l with corresponding MLVSS of 2.64, 2.5 and 2.4 g/l at 50, 100 and 150 mg/l of MG. However, the TC-AS bioreactor showed better removal efficiencies for COD, color, and dye concentration than the AS bioreactor. This may be due to the syntrophic interaction between the AS and *Pseudomonas plecoglossicide* MG2 [32]. The results for MLSS and MLVSS were 3.23, 3.14, 3.6 g/l and 2.51, 2.24, 2.0 g/l for 50, 100 and 150 mg/l, respectively. The MLSS and MLVSS achieved from using the tested culture alone increase with increasing the dye concentration. Increasing the MLSS is attributed to the growth rate of the tested culture; consequently, the increase of MLVSS indicates the growth of *Pseudomonas plecoglossicide* MG2 and this was confirmed by high removal rates of COD and other pollution parameters. The MLSS was 3.19, 4.64, 4.9 g/l with MLVSS of 2.37, 3.5 and 3.6 g/l, respectively for 50, 100 and 150 mg/l.

### *Oxidation reduction potential impact on biodegradation of MG*

The efficiency of pollutant removal can be investigated by measuring the oxidation reduction potential (ORP) of aqueous solutions. ORP represents the redox status of wastewater. There is a linear relationship between COD and ORP. The result showed that as the COD removal efficiency increased as the ORP value increased. This was confirmed by the residual COD and ORP measurements. Moreover, this emphasizes that biodegradation occurs effectively under aerobic conditions [25]. At 50 mg MG /l, the average mean of ORP values of AS and TC-AS bioreactors were 353,352 and 324 mv, respectively. Increasing the concentration of MG up to 100 mg/l, the efficiency of AS bioreactor decreased. However, the behavior of TC bioreactor was the best at higher dye concentrations. The ORP increased from 325 to 432 and 553 at 50, 100 and 150 mg MG/l. In agreement with these results, Li and Bishop [26] indicated that the positive values of ORP emphasized the oxidation process under aerobic conditions and accordingly, increment of COD removal rates with retention time.

### FTIR

**Fig. 12:**
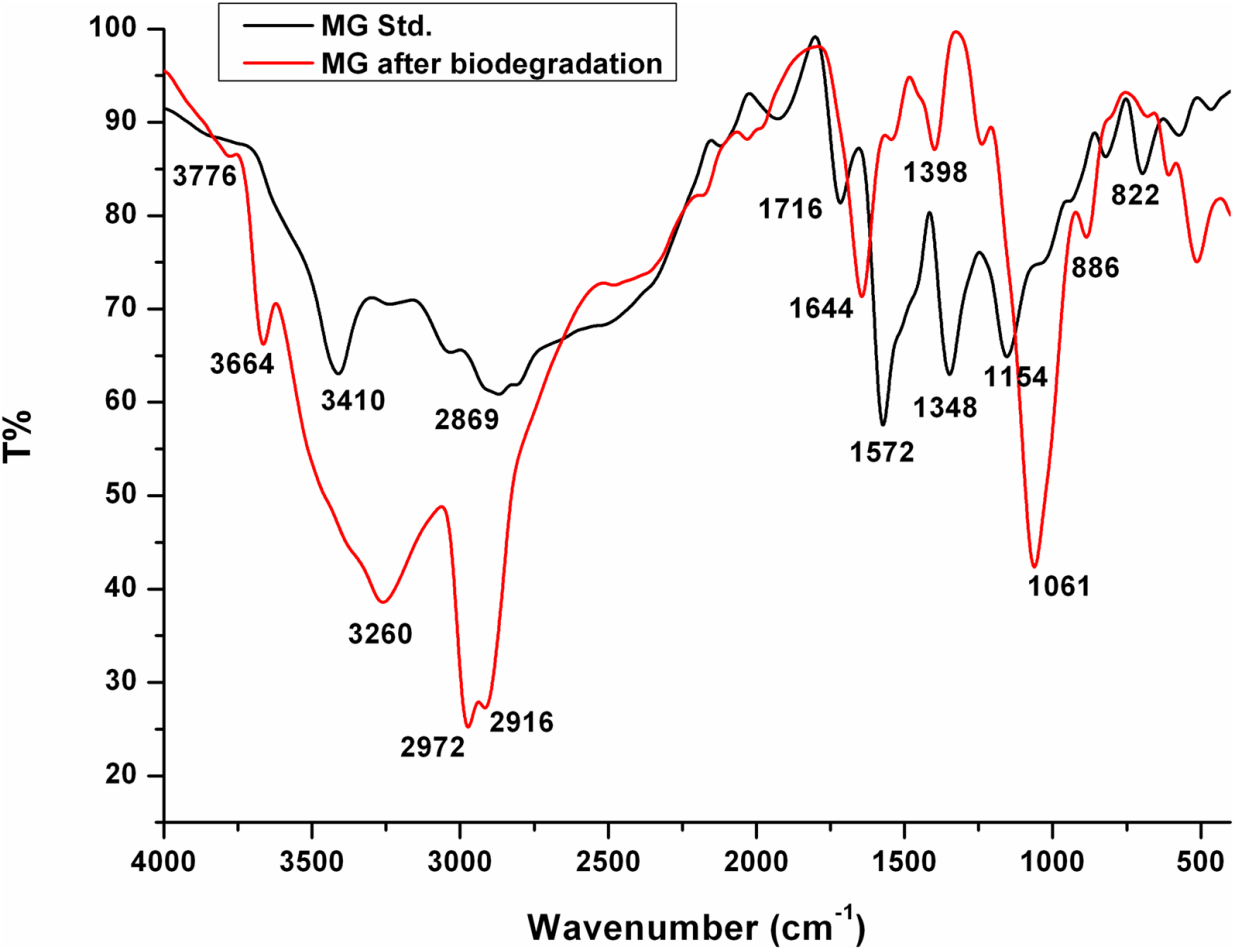
FTIR spectra of MG before and after degradation by *Pseudomonas plecoglossicide* MG2

FTIR spectroscopy is a widely used technique for investigation of dyes degradation. Figure 12 shows the FTIR spectra of MG and its products after biodegradation by *Pseudomonas plecoglossicide* MG2. The spectrum of the untreated MG displayed the main characteristic absorption bands at 3410, 2915, 1572, and 1154 cm^−1^, which are assigned for the vibrations of NH asymmetric stretching, C-H asymmetric stretching, C=C stretching of benzene rings, and the aromatic C-N stretching, respectively. In addition, the peaks at 1348 and 822 cm^−1^ are attributed to the vibrations of –CH_3_ asymmetric bending and C=C trisubstituted benzene rings, respectively.

On the other hand, the spectrum of extracted MG products after biodegradation showed remarkable changes in the fingerprint region 1500–500 cm^−1^. In addition, the disappearance of the characteristic peak of benzene rings at 1572 cm^−1^ indicates to changes in the benzene rings as a result of the biodegradation process. The appearance of new peaks at 3776, 3664 cm^−1^ assigned for OH stretching vibrations due to formation of hydroxylated products [8]. Moreover, another new peak appeared at 1644 cm^−1^, which implies to the formation of C=O during one or more steps of the biodegradation process. The peak at 2916 cm^−1^ is attributed to the –CH stretching by asymmetric CH_2_ group referring to methylene-substituted products [8]. The peak at 1398 cm^−1^ is due to –NH or –CN stretching vibrations in amine III group [8]. A peak at 1239 cm^−1^ for C-N stretch with a sharp peak at 1061 cm^−1^ and a peak around 3260 cm^−1^ for N–H stretch represents the formation of primary and secondary amines. The peak at 886 cm^−1^ of the di-substituted benzene derivatives indicated aromatic nature of amines. Also, the peak at 1716 cm^−1^ that initially appeared in the spectrum of MG before degradation disappeared in the spectrum of degraded MG products. The FTIR analysis of MG degraded products confirms the structural and functional changes in MG during degradation. The FTIR analysis also referred to the formation of hydroxylated and demethylated substituted benzenes as a proposed pathway for MG biodegradation. Similar findings were previously reported for the microbial degradation of MG [5, 6, 8, 9, 12, 37, 38].

### Degradation pathways of MG

#### *LC–ESI–MS analysis of MG degradation products*

**Fig. 13:**
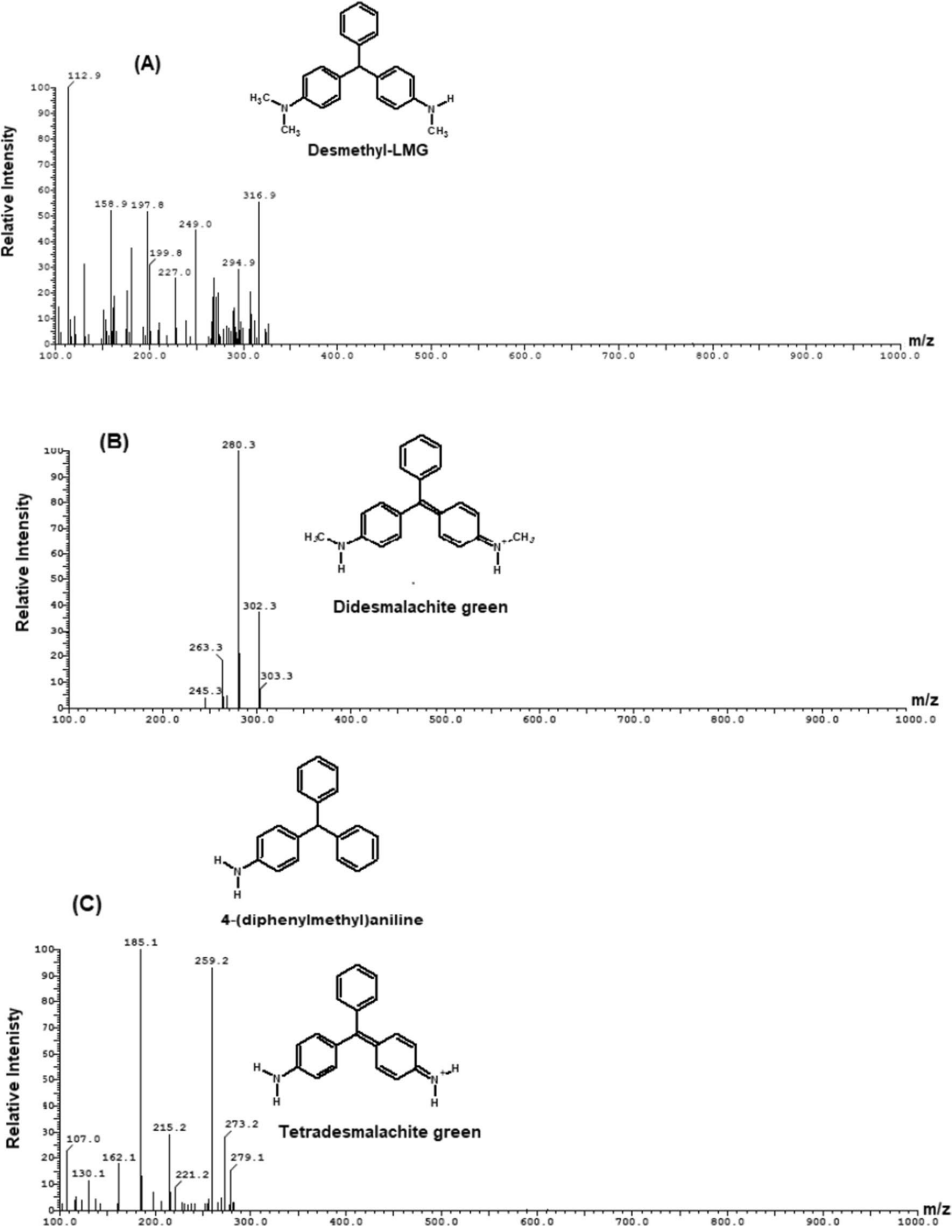

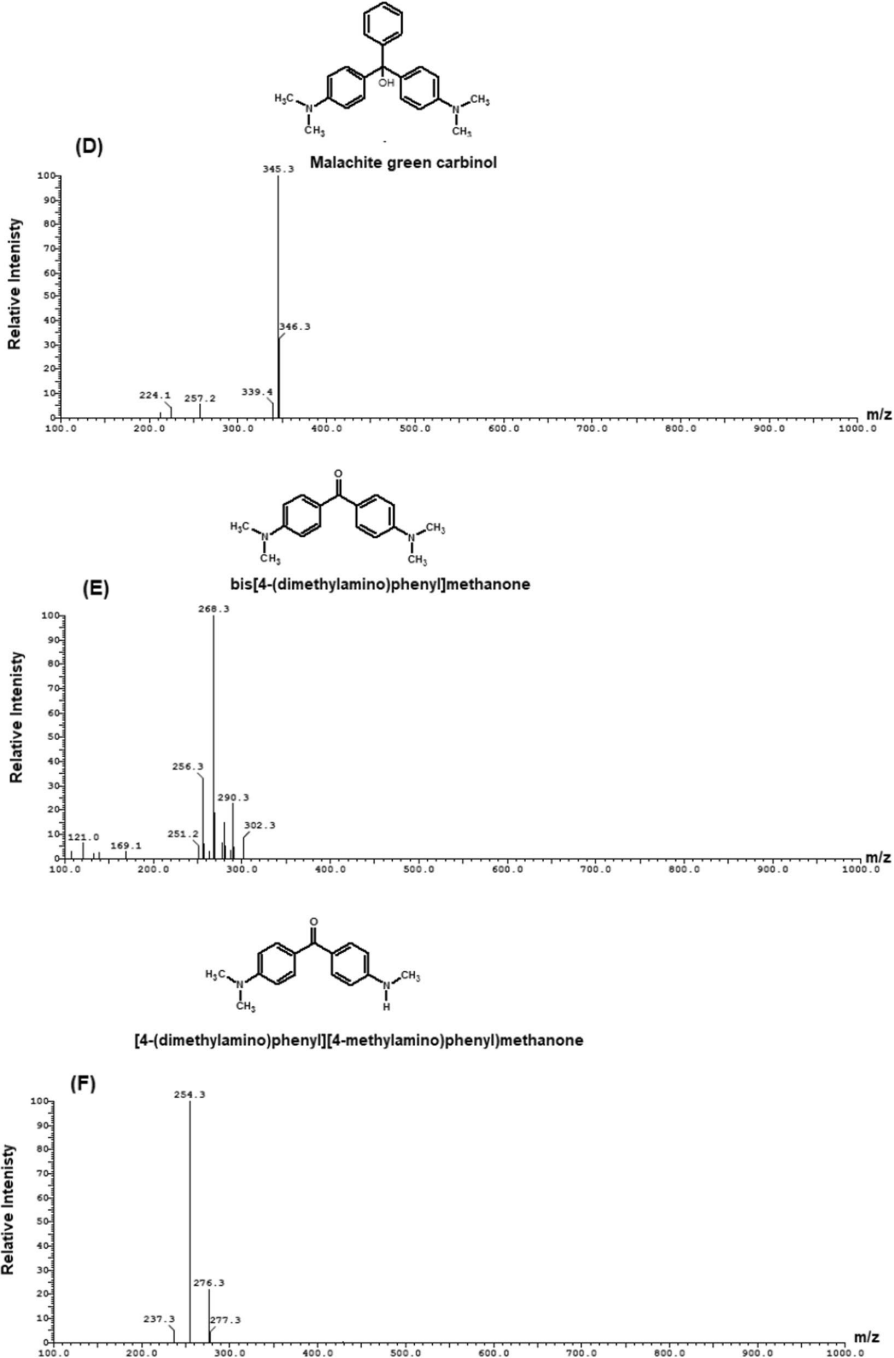

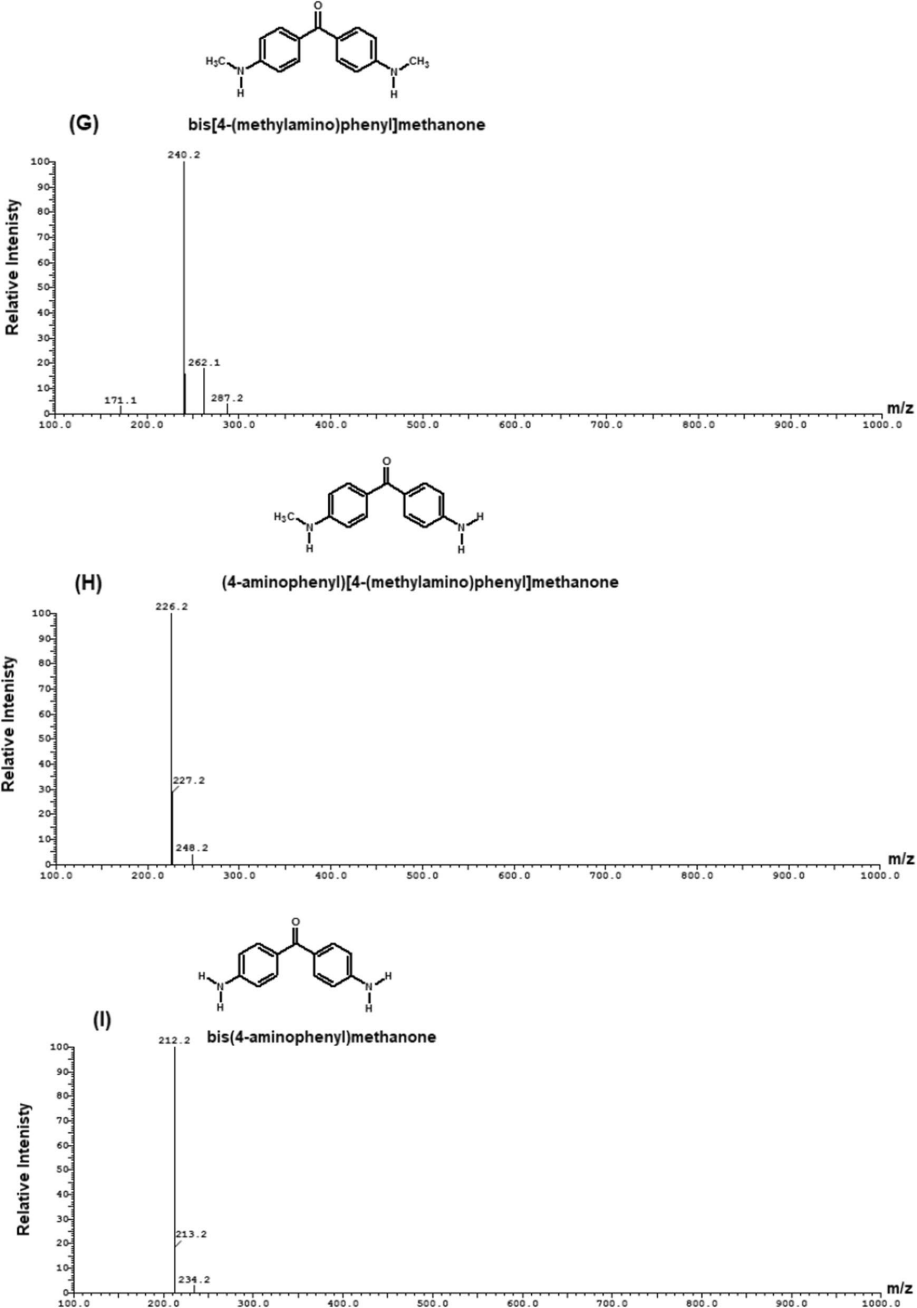

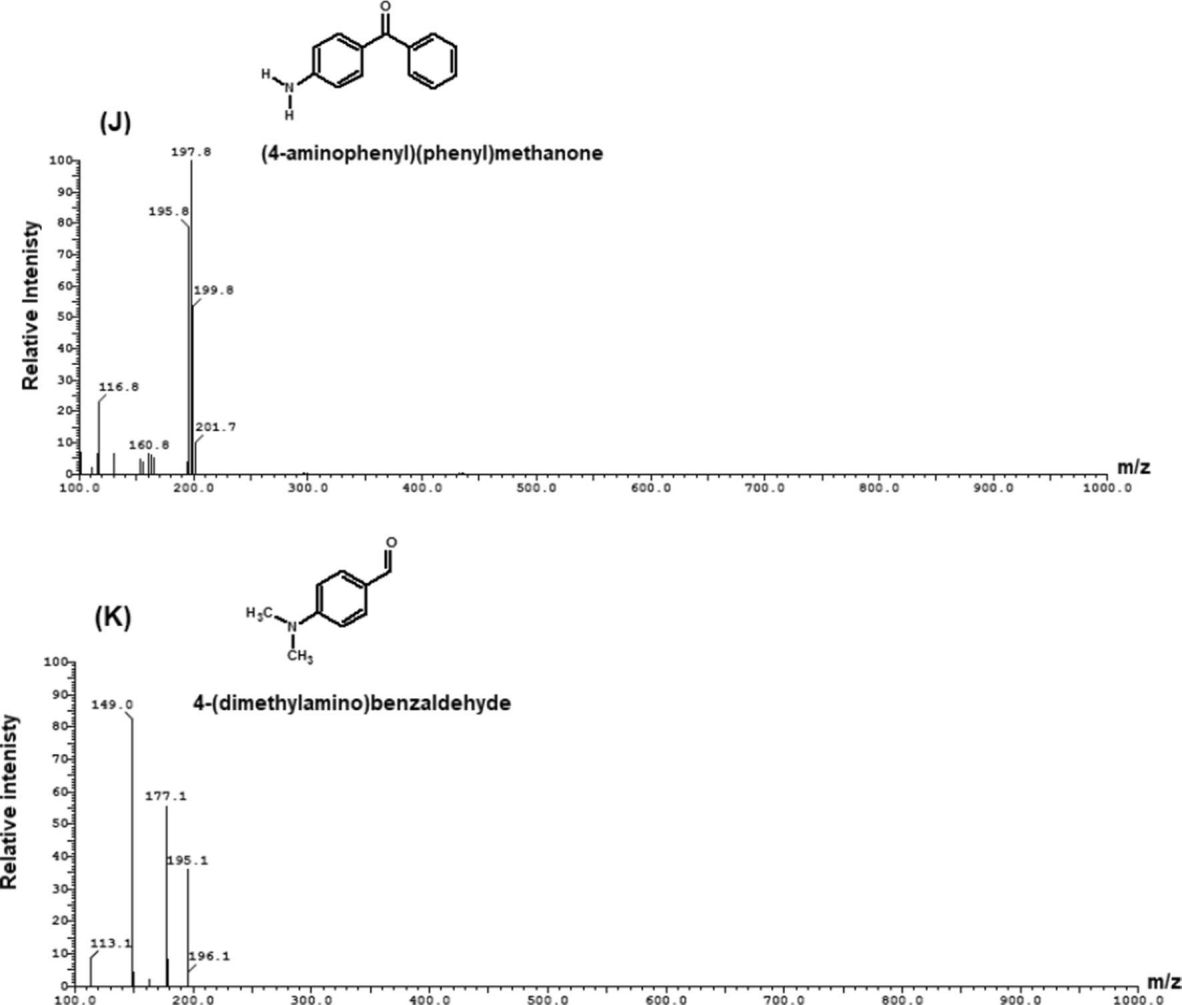
Mass spectra of MG intermediates degradation determined by LC–MS analysis (**A**) desmalachite green (m/z 316, Rt 16.69 min), (**B**) didesmalachite green (m/z 302, Rt 23.39 min), (**C**) tetradesmalachite green (m/z 273, Rt 11.24 min), 4-(diphenylmethyl)aniline (m/z 259, Rt 11.24 min), (**D**) malachite green carbinol (m/z 347, Rt 14.09 min), (**E**) bis[4-(dimethylamino)phenyl]methanone (m/z 268, Rt 23.55 min), (**F**) [4-(dimethylamino)phenyl][4-(methyl-amino)phenyl]methanone (m/z 254, Rt 22.39 min), (**G**) bis[4-(methylamino)phenyl]methanone (m/z 240, Rt 12.47 min), (**H**) (4-amino- phenyl)[4-(methylamino)phenyl]methanone (m/z 226, Rt 15.63 min), (**I**) bis(4-amino phenyl)methanone (m/z 212, Rt 7.26 min), (**J**) (4-amino phenyl)methanone (m/z 197, Rt 5.36 min), and (**K**) 4-(dimathylamino)benzaldehyde (m/z 149, Rt 6.69 min)

The degradation products of MG were determined by LC–ESI–MS analysis and presented in Fig. 13. The results showed that the intermediates of MG degradation were desmalachite green (m/z 316, Rt 16.69 min), didesmalachite green (m/z 302, Rt 23.39 min), tetradesmalachite green (m/z 273, Rt 11.24 min), 4-(diphenylmethyl)aniline (m/z 259, Rt 11.24 min), malachite green carbinol (m/z 347, Rt 14.09 min), bis[4-(dimethylamino)phenyl]methanone (m/z 268, Rt 23.55 min), [4-(dimethylamino)phenyl][4-(methyl-amino)phenyl]methanone (m/z 254, Rt 22.39 min), bis[4-(methylamino)phenyl]methanone (m/z 240, Rt 12.47 min), (4-amino- phenyl)[4-(methylamino)phenyl]methanone (m/z 226, Rt 15.63 min), bis(4-amino phenyl)methanone (m/z 212, Rt 7.26 min), (4-amino phenyl)methanone (m/z 197, Rt 5.36 min), 4-(dimathylamino)benzaldehyde (m/z 149, Rt 6.69 min).

**Fig. 14:**
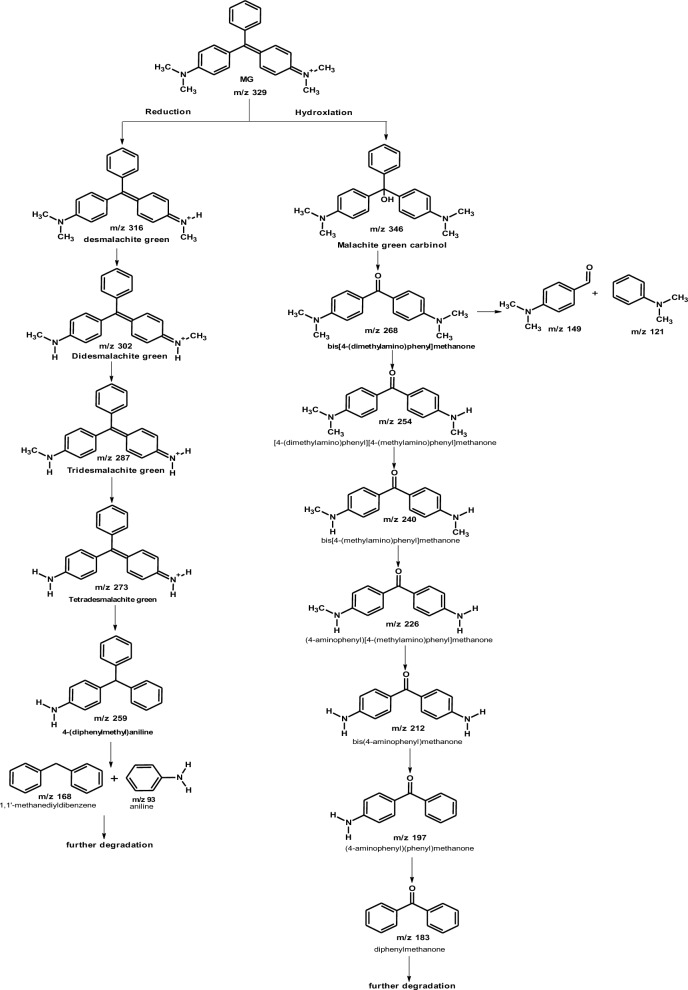
the proposed pathway of MG biodegradation by *Pseudomonas plecoglossicida* MG2

The biodegradation of malachite green by two proposed pathways is shown in Fig. 14. The degradation either directly through a step by step demethylation and hydroxylation process, or indirectly through an oxidative breakdown reaction and a step by step demethylation process. From previous studies, it was concluded that MG biodegradation is initiated by either demethylation process or firstly, reduction reaction and then demethylation process [3]. Similar pathways of triphenylmethane dye degradation by different bacteria were obtained by Ioth et al. [19] who mentioned that the products of crystal violet degradation by *Bacillus subtilis* IF0 13719 and *N. coralline* were diaminophenol and Michler’s ketone. Also, Wang et al. [47] concluded that degradation of MG by *Exiguobacterium* sp. MG2 produced leucomalachite by hydrogenation which is cleaved into 4-dimethylamino-phenyl–phenyl-methanon and subsequently, it is cleaved into 3-dimethylamino-phenol and benzaldehyde.

Abu-Hussien et al. [3] reported that GC–MS and HPLC of the degradation products of MG by *Streptomyces exfoliates* confirmed the presence of leucomalachite green, 1,3-benzenedicarboxylic acid, di-tertbutyl 2- phenylethoxy silane, bis-2- ethylhexyl ester, bis-2-ethylhexyl ester, 1,4-benzenedicarboxylic acid, 1,2-benzenedicarboxylic acid, di-n-octyl phthalate and dioctyl ester. Also, five degradation products could be identified by the degradation of MG by *Pseudomonas veronii* [42]. These intermediate byproducts were leucomalachite green, 4-dimethylamino-benzophenone, 4-dimethyl-aminophenol, hydroquinone and benzaldehyde. Tao et al. [43] found that the degradation of MG by *Pseudomonas* sp. YB2 was confirmed by GC–MS and the degradation intermediates were leucomalachite green, dimethylaniline, 4,4'-bis(dimethylamino) benzophenone, phenol, and 4-(dimethylamino) benzophenone. In another study, LC–MS analysis of biodegradation products of malachite green by *Pseudomonas* sp. strain DY1 confirmed the presence of malachite green carbinol, N,N-di-methylaniline, (dimethyl amino-phenyl)-phenyl-methanone, (amino phenyl)-phenyl methanone, (methyl amino-phenyl)-phenyl-methanone, and di-benzyl methane [13]. In the study of Chaturvedi and Verma [9], GC–MS analysis of the degradation products of MG by *Ochrobactrum pseudogrignonense* strain GGUPV1 showed the presence of 4-(dimethyl amino)pheny-phenyl, phenol-3,5,-demethoxy and phenol-3-(demethylamino).

#### Cytotoxicity of extracted degraded product

Cytotoxicity is one of the most important indicators for biological evaluation in vitro studies. It determines whether a product or a compound will have any toxic effect on living cells. The cytotoxic effect of MG before the treatment with *Pseudomonas plecoglossicide* MG2 on a normal human retina cell line showed a highly toxic effect on vero cells with LC_50_ of 28.9 µg/ml and LC_90_ at 79.7 µg/ml. On the other hand, the extracted products after degradation by *Pseudomonas plecoglossicide* MG2 showed no toxicity on vero cells at all tested concentrations. These data revealed the potential of *Pseudomonas plecoglossicide* MG2 as a MG biodegrader. In accordance with these results, Vilhena et al. [46] found that MG showed high cytotoxicity effect against tested cell lines (ACP02, L929, MNP01, and MRC-5). They also reported that higher MG concentrations exhibited cell necrosis while lower MG concentrations induced opoptosis. In the study of Abu-Hussien et al. [3], they found that MG degradation products (after degradation by *Streptomyces exfoliates*) exhibited no cytotoxicity on human skin fibroblast normal cells (HSF).

## Conclusion

In this study, an isolated *Pseudomonas plecoglossicide* MG2 from the sludge of dye industry effluent efficiently degraded the MG at a wide range of pH and at room temperature under static and shaking conditions. Also, it showed a high performance in MG degradation in the stimulated wastewater bioreactors. The degradation products were analysed by LC–MS and FTIR and they showed no cytotoxicity at all tested concentrations. In addition, MG degradation pathways by tested organism was proposed. Subsequently, this study revealed that *Pseudomonas plecoglossicide* MG2 is an efficient cost-effective, and feasible tool for bioremediation of dyes wastewater which represents a seriousness on human and environment. In the near future, an effort will be made for transferring the MG degradation technology from lab to the environmental application sites.

## Data Availability

The datasets generated and/or analyzed during the current study are available from the corresponding author on reasonable request.
